# Prediction of neonatal morbidity and very preterm delivery using maternal steroid biomarkers in early gestation

**DOI:** 10.1371/journal.pone.0243585

**Published:** 2021-01-06

**Authors:** Avinash S. Patil, Chad A. Grotegut, Nilesh W. Gaikwad, Shelley D. Dowden, David M. Haas

**Affiliations:** 1 Department of Obstetrics and Gynecology, University of Arizona College of Medicine-Phoenix, Phoenix, Arizona, United States of America; 2 Valley Perinatal Services, Phoenix, Arizona, United States of America; 3 Department of Obstetrics and Gynecology, Wake Forest University, Winston-Salem, North Carolina, United States of America; 4 Gaikwad Steroidomics Laboratory, Davis, California, United States of America; 5 Department of Obstetrics and Gynecology, Indiana University School of Medicine, Indianapolis, Indianapolis, United States of America; 6 Division of Clinical Pharmacology, Department of Medicine, Indiana University School of Medicine, Indianapolis, Indianapolis, United States of America; University of Liverpool, UNITED KINGDOM

## Abstract

**Background:**

Preterm delivery is a common pregnancy complication that can result in significant neonatal morbidity and mortality. Limited tools exist to predict preterm birth, and none to predict neonatal morbidity, from early in pregnancy. The objective of this study was to determine if the progesterone metabolites 11-deoxycorticosterone (DOC) and 16-alpha hydroxyprogesterone (16α-OHP), when combined with patient demographic and obstetric history known during the pregnancy, are predictive of preterm delivery-associated neonatal morbidity, neonatal length of stay, and risk for spontaneous preterm delivery prior to 32 weeks’ gestation.

**Methods and findings:**

We conducted a cohort study of pregnant women with plasma samples collected as part of Building Blocks of Pregnancy Biobank at the Indiana University School of Medicine. The progesterone metabolites, DOC and 16α-OHP, were quantified by mass spectroscopy from the plasma of 58 pregnant women collected in the late first trimester/early second trimester. Steroid levels were combined with patient demographic and obstetric history data in multivariable logistic regression models. The primary outcome was composite neonatal morbidity as measured by the Hassan scale. Secondary outcomes included neonatal length of stay and spontaneous preterm delivery prior to 32 weeks’ gestation. The final neonatal morbidity model, which incorporated antenatal corticosteroid exposure and fetal sex, was able to predict high morbidity (Hassan score ≥ 2) with an area under the ROC curve (AUROC) of 0.975 (95% CI 0.932, 1.00), while the model without corticosteroid and fetal sex predictors demonstrated an AUROC of 0.927 (95% CI 0.824, 1.00). The Hassan score was highly correlated with neonatal length of stay (p<0.001), allowing the neonatal morbidity model to also predict increased neonatal length of stay (53 [IQR 22, 76] days vs. 4.5 [2, 31] days, above and below the model cut point, respectively; p = 0.0017). Spontaneous preterm delivery prior to 32 weeks’ gestation was also predicted with an AUROC of 0.94 (95% CI 0.869, 1.00).

**Conclusions:**

Plasma levels of DOC and 16α-OHP in early gestation can be combined with patient demographic and clinical data to predict significant neonatal morbidity, neonatal length of stay, and risk for very preterm delivery, though validation studies are needed to verify these findings. Early identification of pregnancies at risk for preterm delivery and neonatal morbidity allows for timely implementation of multidisciplinary care to improve perinatal outcomes.

## Introduction

Premature birth less than 32 weeks’ gestation (very or extremely preterm delivery, vePTD) is associated with exceptionally high rates of neonatal morbidity and associated healthcare expenses [1]. In the United States, vePTD is linked to an 80-fold increase in infant mortality compared to women delivering at term [2]. Despite technological advances, the rate of early preterm delivery (<32 weeks’ gestation), which accounts for the majority of infant mortality, remains unchanged [3]. The lack of progress in reducing the rate of early preterm birth has hampered efforts to improve downstream sequelae, including disparities in infant mortality rates.

Maternal stress has been recognized as an etiology of approximately 60% of preterm births and disproportionately affects African-American women [4]. Activation of the maternal/fetal hypothalamic-pituitary-adrenal (HPA) axis has been associated with maternal stress responses and may lead to a molecular cascade resulting in preterm labor. An imbalance of progesterone metabolism into mineralocorticoid and glucocorticoid pathways has also been linked to an increased risk of vePTD and may be a manifestation of the maternal stress phenotype [5]. In particular, the molecules 11-deoxycorticosterone (DOC) and 16α-hydroxyprogesterone (16α-OHP) allow early identification of women at risk for vePTD when measured during the late first trimester/early second trimester [5].

Infants born very preterm exhibit a deficit in adrenal steroid synthesis resulting in a sodium wasting phenomenon [6]. This relative adrenocortical insufficiency has been associated with an increased risk of neonatal bronchopulmonary dysplasia or death [7]. The diminished production of endogenous steroids has been attributed to adrenal immaturity inversely correlated to gestational age at delivery. Recently, untargeted metabolomic analysis of amniotic fluid has been used to identify pregnancies at risk of spontaneous preterm delivery [8]. Furthermore, in this study, different amniotic fluid metabolic profiles of endogenous steroids and fatty acids existed among those preterm deliveries complicated with neonatal bronchopulmonary dysplasia from those preterm deliveries without neonatal bronchopulmonary dysplasia. Despite these interesting findings, amniotic fluid is difficult to obtain for routine diagnostic purposes.

Significant overlap exists between birth at an early gestational age and neonatal morbidity [9]. Aberration in adrenal steroid production appears to be a common feature in gravida who subsequently have vePTD and in neonates with sequelae of prematurity. The molecules DOC and 16α-OHP have been associated with vePTD when measured from the late first trimester/early second trimester [5]. The objective of this study was to determine if obstetric and demographic variables known during the pregnancy, when combined with steroid metabolite biomarkers (DOC and 16α-OHP) obtained early in pregnancy, could predict the risk of preterm delivery-associated neonatal morbidity in a low risk population of pregnant women. A secondary objective of the study was to optimize the ability to predict preterm delivery prior to 32 weeks beyond that provided by the biomarkers alone [5]. We hypothesized that a similar adrenal steroid synthesis profile exists in women with vePTD and neonates with sequelae of prematurity; thus, modeling of DOC and 16α-OHP concentrations in combination with patient demographic and clinical variables would allow for prediction of neonatal morbidity and vePTD from early in pregnancy.

## Materials and methods

Targeted steroid metabolomics was performed on prospectively collected plasma specimens collected from women participating in an academic medical center pregnancy biobank to investigate the ability of DOC and 16α-OHP to predict neonatal morbidity and vePTD. The Building Blocks of Pregnancy Biobank at the Indiana University School of Medicine (IU BBPB) is a longitudinal, prospective pregnancy biorepository with a range of maternal specimens collected through convenience sampling every trimester, in addition to clinical data on current and prior obstetric outcomes. Plasma samples from women enrolled in the biobank with a singleton gestation that were collected from the late first trimester/early second trimester were utilized. Specimens were obtained from all available subjects who delivered very preterm and moderate-late preterm [1]. Specimens available in the biobank that originated from subjects that delivered at term were matched to preterm specimens by BMI. Samples from subjects from each group (preterm and term) were excluded if subjects used steroids or anticoagulants prior to the sample being collected during the pregnancy. The study was approved by the Indiana University Institutional Review Board (IRB, IRB #1011003384) and all subjects provided written informed consent.

A targeted metabolomics approach, developed at Gaikwad Steroidomics Laboratory, was used to quantify endogenous 11-deoxycorticosterone (DOC) and 16α-hydroxyprogesterone (16α-OHP) using ultraperformance liquid chromatography-tandem mass spectrometry (UPLC/MS-MS) analysis. The frozen human plasma samples were processed using an assay previously validated on the MS platform [10, 11]. Briefly, 1–2 mL aliquots of plasma were adjusted to pH 7.0 and subject to solid phase extraction with methanol. Analytical separations of the methanol fraction on the UPLC system were conducted with C18 or phenyl columns (1 X 100 mm) at a flow rate of 0.15 ml/min. The gradient was started with 100% A (0.1% formic acid in H_2_O) and 0% B (0.1% formic acid in CH_3_CN), changed to 80% A over 10 min, followed by a 10-min linear gradient to 0% A, resulting in a total separation time of 20 min. The elutions from the UPLC column were introduced to the mass spectrometer. All MS experiments were performed using electrospray ionization (ESI) in positive ion and negative ion mode, with an ESI-MS capillary voltage of 3.0 kV, an extractor cone voltage of 2 V, and a detector voltage of 650 V. The following MS conditions were used: desolvation gas at 600 l/h, cone gas flow at 60 l/h, desolvation temperature at 200 °C, and source temperature 100 °C. Pure standards were used to optimize the UPLC-MS/MS conditions, make calibration curves, and generate the multiple reaction monitoring method (MRM). The resulting data was processed using MassLynx 4.1 software (Waters Corporation, Milford, MA, USA).

Regression models were developed to predict the likelihood of neonatal morbidity (neonatal morbidity model) and the likelihood of spontaneous delivery prior to 32 weeks’ gestation (vePTD model). In addition to the maternal plasma concentrations of DOC and 16α-OHP, the influence of several pre-determined maternal demographic and obstetric variables that are known during the pregnancy were evaluated: prior preterm birth, subject age, ethnicity/race, body mass index, the number of prior preterm births, the number of prior miscarriages, sex of the baby, and antenatal corticosteroid use. These variables were selected as they may potentially affect the risk for adverse neonatal morbidity or preterm delivery. The neonatal model was constructed first with only information known at the onset of pregnancy, thus fetal sex and antenatal corticosteroid use were excluded as predictors. A second neonatal model was also constructed including fetal sex and antenatal corticosteroid use as relevant information that becomes available as gestation progresses. The dependent variable for the neonatal morbidity model was a binary measure of low or high neonatal morbidity. The Hassan scale was used to generate a composite neonatal morbidity score; the scale is scored between zero and four based on the number of major sequelae of prematurity or death in the neonatal period [12]. The sequelae of prematurity that make up the composite scale include respiratory distress syndrome, retinopathy, bronchopulmonary dysplasia, intraventricular hemorrhage, periventricular leukomalacia, sepsis, or necrotizing enterocolitis. Each subject was binarized into low (0–1) and high (2–4) neonatal morbidity groups based on their Hassan score. The dependent variable for the vePTD model was spontaneous delivery prior to 32 weeks (Yes/No).

Stepwise binary logistic regression modeling was performed using R software version 3.6.2 (R Foundation for Statistical Computing). Samples size calculations for multiple regression were based on α = 0.05, 80% power, and an anticipated effect size of 0.5. A large effect size was expected based on the results of our earlier study [5]. Considering the maximum of nine predictors utilized in the model, a minimum sample size of 41 would be required. To avoid over-fitting, the Akaike Information Criterion (AIC) and R^2^ values were used to identify the optimal model. Differences in neonatal length of stay were compared using the Mann-Whitney test among those women that screened positive with the final neonatal morbidity model compared to those women who screened negative with the final neonatal morbidity model. Whether a woman screened positive or negative with the neonatal morbidity model was based on the cut point in ROC analysis that lead to the best area under the curve. Finally, the Hassan score for each subject was correlated with the number of days the subject’s neonate was initially hospitalized following birth using an ANOVA test for linear trend. Statistical analyses were performed using R software version 3.6.2 (R Foundation for Statistical Computing) and GraphPad Prism version 8.3.1 (GraphPad Software, San Diego, CA).

## Results

Plasma samples from fifty-eight subjects were available for biomarker analysis with complete associated data. The median (IQR) gestational age that plasma samples were collected was 16 (13, 17) weeks’ gestation. Among the fifty-eight included subjects, 40 delivered preterm (less than 37 weeks) and 18 delivered at term (greater than or equal to 37 0/7 weeks). The median (IQR) of women delivering preterm (less than 37 weeks) and term were 33 (28, 34) and 39 (38, 39) weeks’ gestation, respectively. Within the preterm group, 17 delivered less than 32 weeks and of these, 11 delivered spontaneously less than 32 weeks’ gestation. The median (IQR) of women delivering less than 32 weeks was 27 (22.5, 30.5) weeks’ gestation. Ten subjects had a Hassan score of 2 or higher, with the remaining forty-eight subjects having Hassan scores of 0–1. Subject characteristics by Hassan score are described in Table 1. Women delivering babies with an elevated Hassan score of 2–4 were more likely to have a higher BMI and deliver at a lower gestational age than those women delivering a baby with a low Hassan score (Table 1). Neonates with an elevated Hassan score were born at a lower gestational age and had a lower associated birthweight, were more likely to receive antenatal corticosteroids, have a lower five-minute Apgar score, and require resuscitative measures at birth compared to babies having a low Hassan score (Table 1).

**Table 1:** Subject demographics within low (Hassan score 0–1) and high (Hassan score 2–4) neonatal morbidity groups.

	Hassan 0–1 (n = 48)	Hassan 2–4 (n = 10)	p-value
***Maternal***			
Age, years	24 (20–29)	27.5 (19–31)	0.73
Race/Ethnicity			
White	21 (44)	2 (20)	0.41
Black	20 (42)	7 (70)	
Hispanic	6 (12)	1 (10)	
Asian	1 (2)	0 (0)	
BMI, kg/m^2^	28.2 (22.9–35)	37.8 (30.3–44.4)	0.02
Gravidity	2 (1–3)	1 (1–2)	0.13
Diabetes mellitus	5 (10)	0 (0)	0.29
Chronic hypertension	7 (15)	2 (20)	0.67
History of preterm delivery	9 (19)	0 (0)	0.14
sPTD <32 weeks, current pregnancy	5 (10)	6 (60)	<0.001
Mode of delivery			
Spontaneous vaginal	22 (46)	6 (60)	0.74
Cesarean	22 (46)	4 (40)	
Assisted vaginal	2 (4)	0 (0)	
VBAC	2 (4)	0 (0)	
Induction of labor	10 (21)	1 (10)	0.43
***Neonatal***			
Gestational age at birth, weeks	35 (34–39)	27 (22–29)	<0.001
BTMZ utilization	11 (23)	8 (80)	0.001
5-minute Apgar score	9 (8–9)	7 (2–7)	<0.001
Baby sex			
Female	25 (52)	4 (40)	0.49
Male	23 (48)	6 (60)	
Birth weight, grams	2597 (2163–3280)	1120 (573–1498)	<0.001
Hospitalization, days	6 (2–30)	72 (33–78)	0.001
Neonatal resuscitative measures at delivery	14 (29)	9 (90)	<0.001
Ventilatory support	6 (13)	3 (30)	0.16
Surfactant use	5 (10)	4 (40)	0.02
Antibiotic use	17 (35)	7 (70)	0.04
RDS	16 (33)	8 (80)	0.006
Sepsis	0 (0)	4 (40)	<0.001
Necrotizing enterocolitis	0 (0)	2 (20)	0.002
IVH	0 (0)	2 (20)	0.002

Values are median (IQR) or number of subjects, n (%).

*Abbreviations*: BMI, body mass index; BTMZ, betamethasone; IVH, intraventricular hemorrhage; RDS, respiratory distress syndrome; sPTD, spontaneous preterm delivery; VBAC, vaginal birth after cesarean

**Fig 1:**
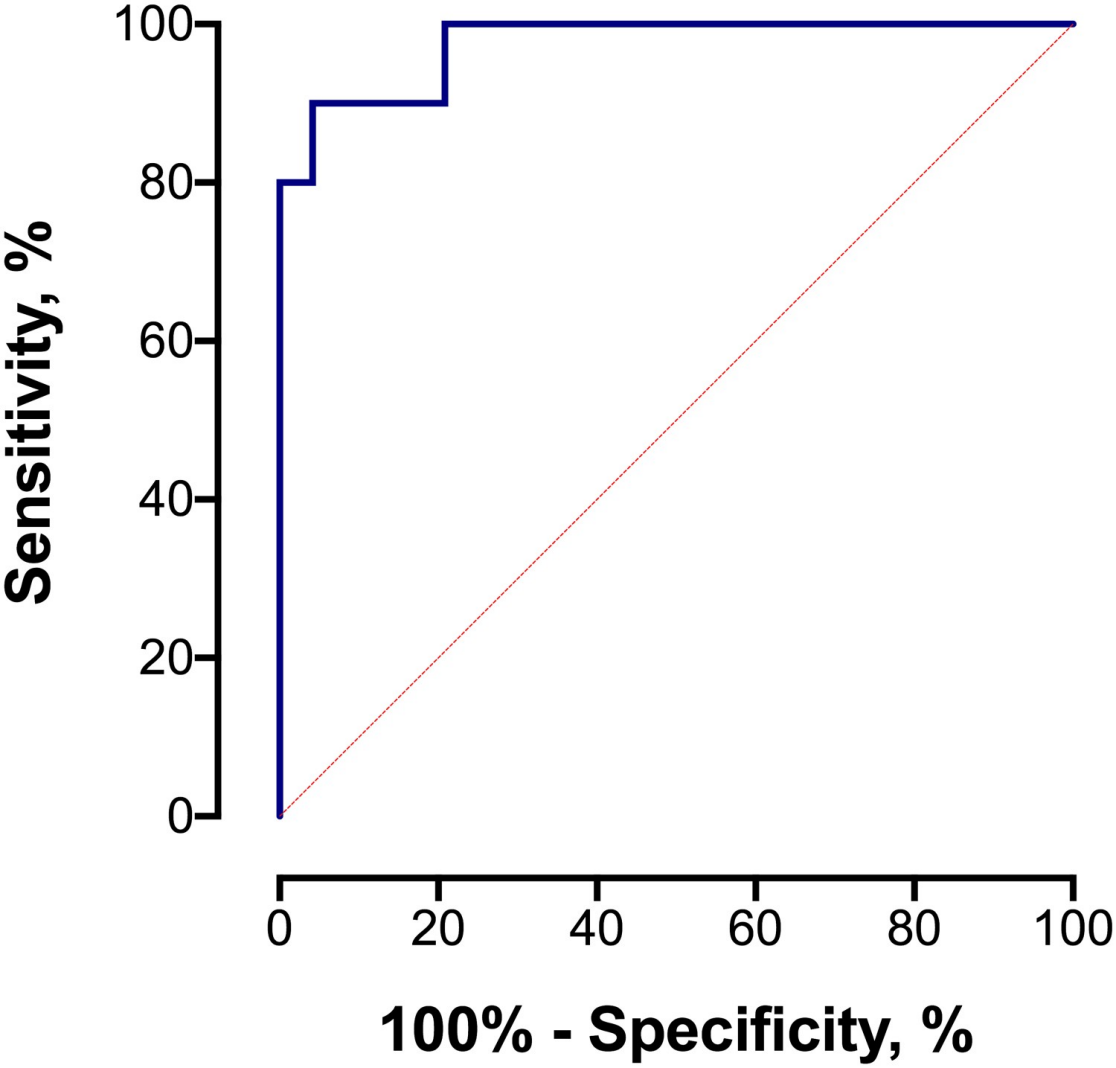
Neonatal morbidity model ROC curve, incorporating biomarkers and demographic and clinical characteristics. The optimal neonatal morbidity model, which included the biomarkers and demographic and clinical characteristics including whether antenatal corticosteroids had been given and fetal sex, demonstrated an area under the ROC curve of 0.975 (95% CI 0.932, 1.00) for discriminating subjects with a Hassan score of 2–4 (high neonatal morbidity) from those with a Hassan score of 0–1 (none or low neonatal morbidity). At this optimal cutoff value, the corresponding sensitivity was 90% and the specificity was 96%, with a corresponding +LR of 21.60 (95% CI 5.48, 85.21) for women with a positive result.

**Fig 2:**
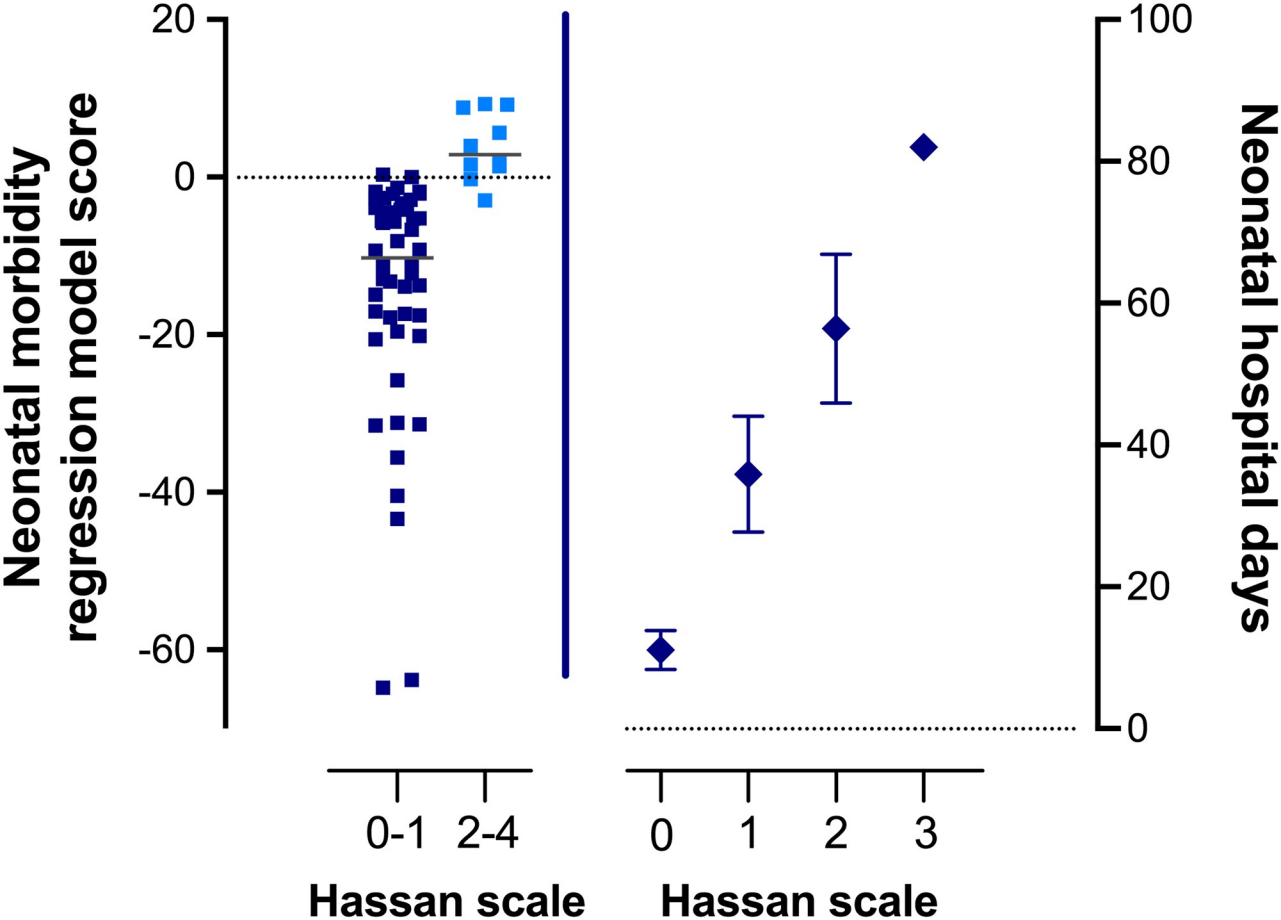
Neonatal morbidity regression output and neonatal length of stay associated with neonatal morbidity composite scale. The regression outputs for the neonatal morbidity model (left panel) differed significantly (unpaired, non-parametric t-test p-value <0.001) by Hassan composite neonatal score of 0–1 (low neonatal morbidity score) compared to 2–4 (high neonatal morbidity score). The horizontal bars represent the median regression output score for each group. The Hassan composite score strongly correlated with number of neonatal hospital days (one-way ANOVA test for linear trend p<0.001) between a Hassan score of 0 and up to a Hassan score of 3 (right panel). Values are mean with error bars representing the standard error of the mean.

The performance of DOC and 16α-OHP alone for predicting neonatal morbidity was determined with a logistic regression model to be R^2^ = 9.3%. Stepwise logistic regression identified the covariates age, race/ethnicity, BMI, the number of prior miscarriages, antenatal corticosteroids use, and sex of the baby as significantly contributing to the model with acceptable standard error. The model was first run without the fetal sex and antenatal corticosteroid predictors, and then again with both predictors. Without the fetal sex and antenatal corticosteroid predictors, the neonatal morbidity multivariable model predicted a Hassan score of 2 or higher with an R^2^ of 56%, and with both predictors included an R^2^ of 75%. The neonatal morbidity model without fetal sex and antenatal corticosteroid predictors achieved an area under the receiver operating characteristic curve (AUROC) of 0.927 (95% CI 0.824, 1.00) with optimal sensitivity of 90% and specificity of 90% (S1 Fig). Inclusion of fetal sex and antenatal corticosteroid predictors improved the AUROC to 0.975 (95% CI 0.932, 1.00) with optimal sensitivity (90%) and specificity (96%) (Fig 1). The final model characteristics included a positive predictive value (PPV) of 0.82 and negative predictive value (NPV) of 0.98. The distribution of the model’s logistic regression scores illustrates a significant differentiation between subjects with Hassan score of 0–1 compared to those subjects with a Hassan score of 2–4 (p<0.001, Fig 2 left panel).

To determine the impact of the biomarkers to the model we compared the Likelihood Ratio (LR) for neonatal morbidity in women with a positive test result within the various models. The +LR for a Hassan Score of 2–4 based on the clinical covariates alone, without fetal sex and corticosteroid administration, was 1.77 (95% CI 1.04, 3.02). The addition of the DOC and 16α-OHP biomarkers to this model increased the +LR to 8.64 (95% CI 3.67, 20.32) for having a Hassan Score of 2–4 with a positive test result. Finally, when fetal sex and corticosteroid administration was added to the final model (clinical covariates including fetal sex and corticosteroid administration) to the biomarkers, the +LR for neonatal morbidity as measured with a Hassan Score of 2–4 was 21.60 (95% CI 5.48, 85.21) for women with a positive test result.

**Fig 3:**
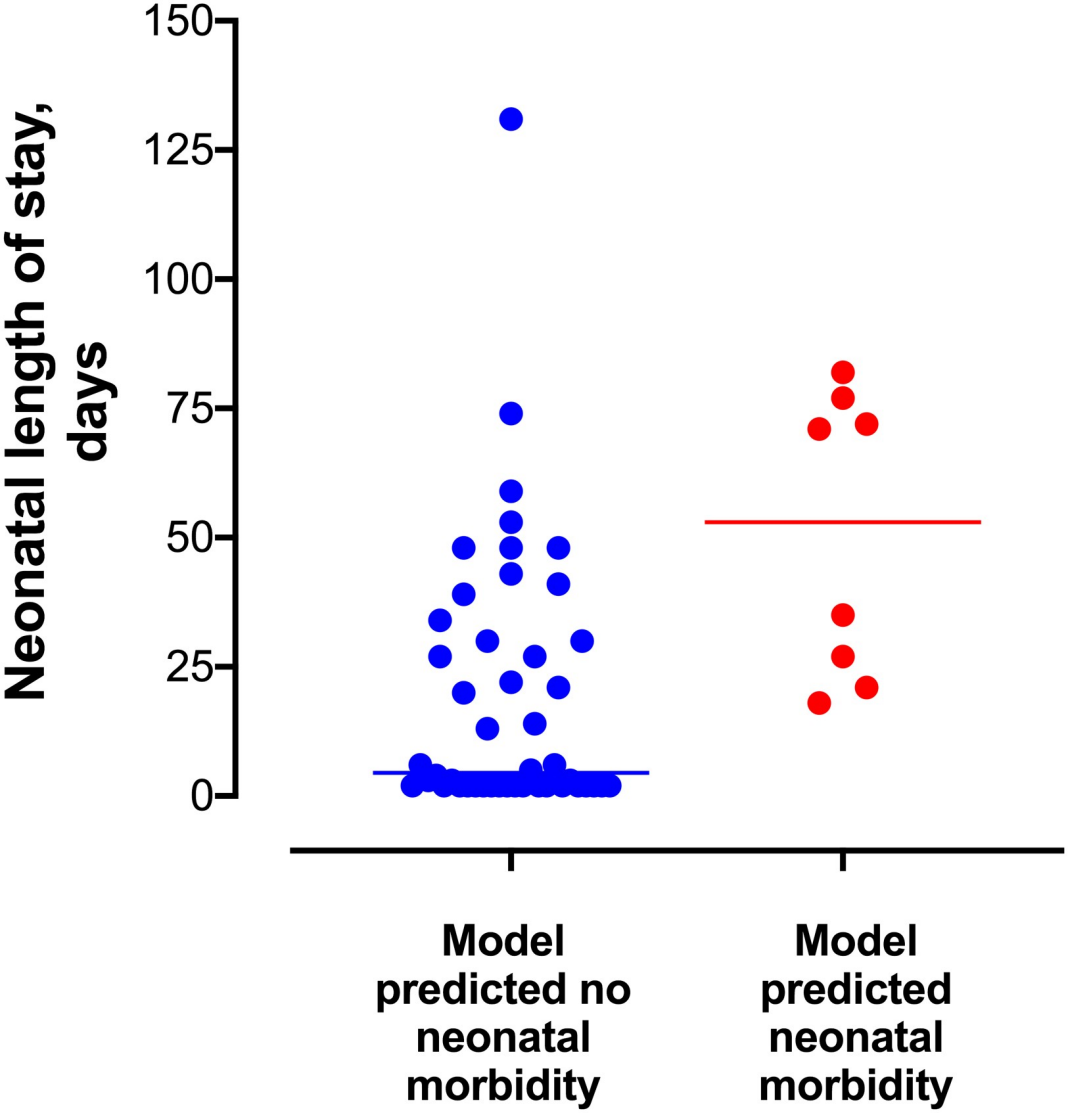
Neonatal length of stay categorized by results of the final neonatal morbidity predictive model. The median (IQR) length of neonatal hospitalization differed significantly between women who screened positive with the neonatal morbidity model compared to those women who screened negative (53 [IQR 22.5, 75.7] days vs. 4.5 [2, 31] days, p = 0.0017). The horizontal bars represent the median neonatal length of stay.

Neonatal length of stay, or the number of days that a newborn was admitted to the hospital following birth, was compared between those women who screened positive with the final logistic regression model for neonatal morbidity compared to those women who screened negative. Neonates of women who screened positive with the neonatal morbidity model had significantly longer median length of hospital stays compared to those women who screened negative (53 [IQR 22.5, 75.7] days vs. 4.5 [2, 31] days, respectively; p = 0.0017, Fig 3). The Hassan composite score also strongly correlated with number of neonatal hospital days, with an incremental increased median length of stay from a Hassan score of 0 up through a score of 3 (p<0.001) (Fig 2 right panel). There were four women whose neonates died following delivery (Hassan score 4). Three of the four (75%) neonates died on the day of birth (length of stay = 0) and the fourth child had a length of stay of 28 days prior to death. Given that the majority of neonates with a Hassan score of 4 (mortality) died on the day of delivery and as such their length of stay was zero, those neonates with a Hassan score of 4 were not included in the neonatal length of stay calculations (Fig 2 right panel and Fig 3).

**Fig 4:**
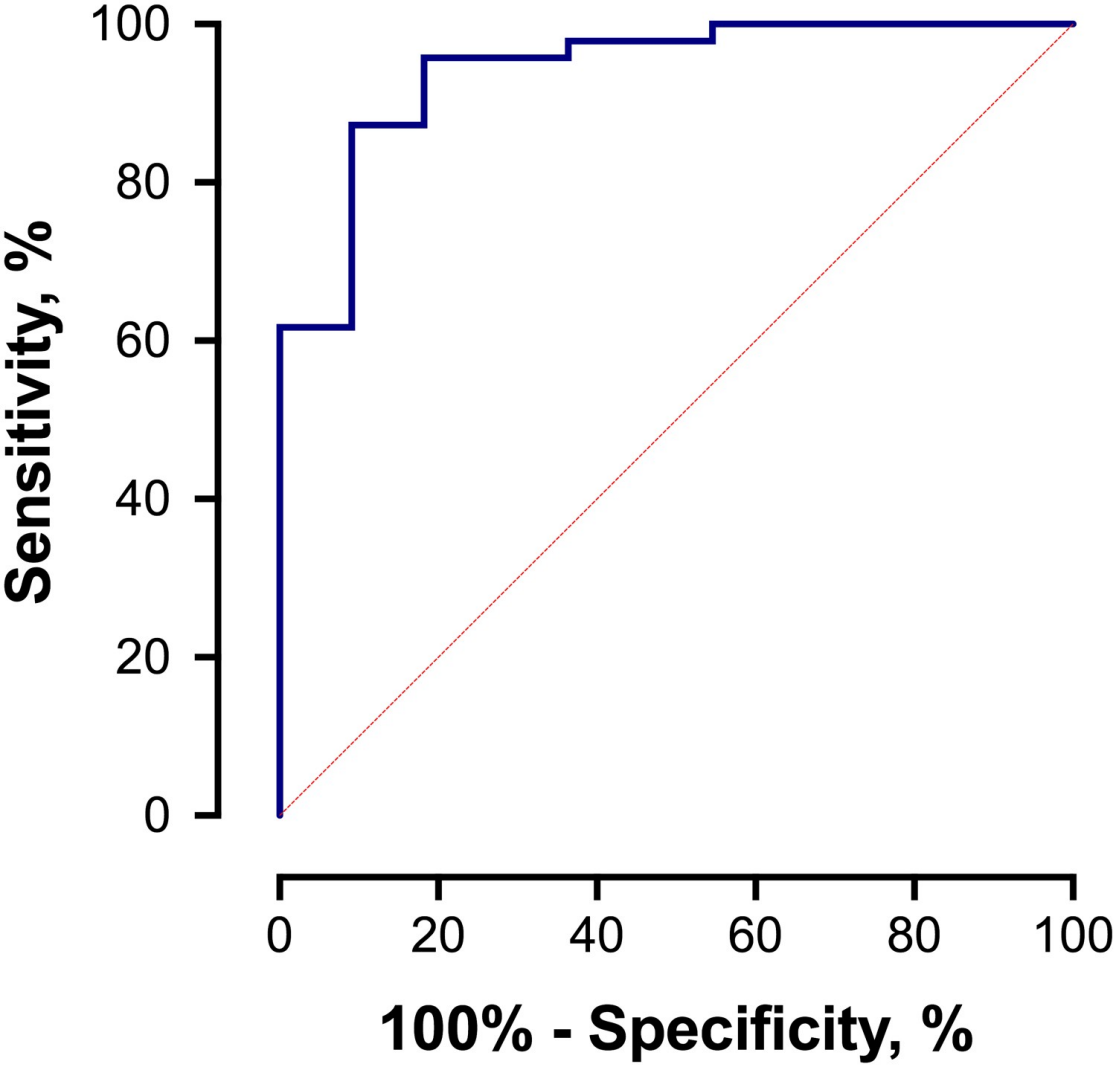
Preterm birth model ROC curve, incorporating biomarkers and demographic and clinical characteristics. The optimal preterm model demonstrated an area under the ROC curve of 0.942 (95% CI 0.869, 1.00) for discriminating subjects delivering spontaneously less than 32 weeks from women having either medically-indicated deliveries prior to 32 weeks, or those women having any delivery at or greater than 32 weeks. At this optimal cutoff value, the sensitivity was 91% and the specificity was 87%, resulting in a +LR for spontaneous preterm birth less than 32 weeks’ gestation of 7.12 (95% CI 3.30, 15.39) for women with a positive test result.

**Fig 5:**
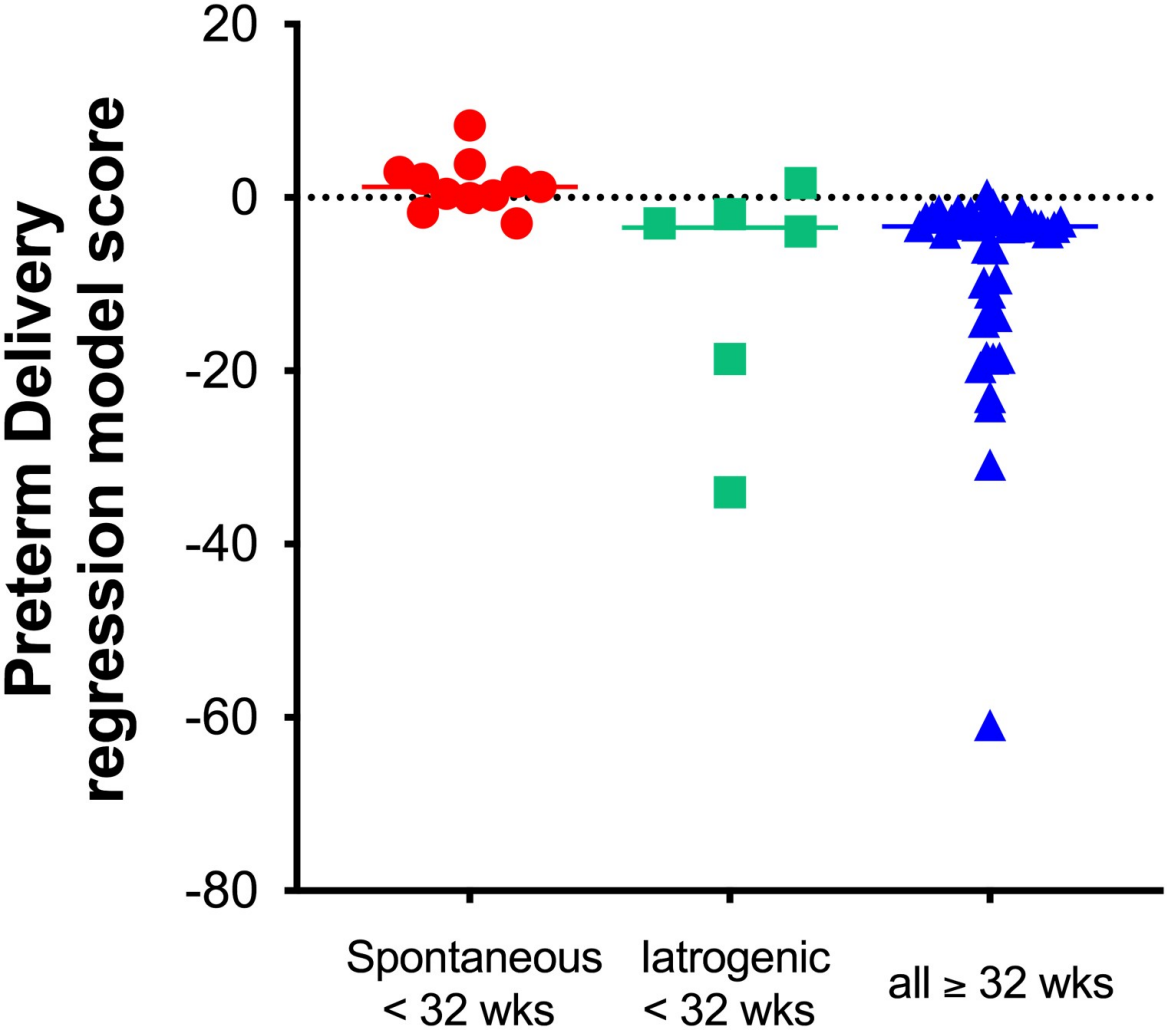
Regression output and preterm birth phenotype. The regression outputs differed significantly (ANOVA p-value = 0.018) by birth phenotype; spontaneous preterm delivery less than 32 weeks, iatrogenic preterm delivery less than 32 weeks, and all deliveries at or greater than 32 weeks. The horizontal bars represent the median regression output score for each group.

Our prior work demonstrated that the performance of the DOC and 16α-OHP biomarkers alone for predicting spontaneous preterm delivery prior to 32 weeks resulted in a model R^2^ = 11% [5]. In the current study, stepwise logistic regression identified the covariates parity, age, race, BMI, prior preterm deliveries, and prior miscarriages as significant for the preterm delivery model, leading to improvements in R^2^ to 59% in the multivariable model for prediction of spontaneous preterm delivery prior to 32 weeks’ gestation. This enhanced preterm delivery model’s AUROC was 0.942 (95% CI 0.869, 1.00) with optimal sensitivity of 91% and specificity of 87% (PPV = 0.63, NPV = 0.98), compared with an AUROC of 0.805 (95% CI 0.644, 0.965) with sensitivity 89% and specificity 59% when the biomarkers were used alone (Fig 4) [5]. Fig 5 demonstrates the distribution of the enhanced final model’s logistic regression scores by delivery phenotype; spontaneous preterm delivery less than 32 weeks, iatrogenic/medically-indicated preterm delivery less than 32 weeks, and all deliveries at or greater than 32 weeks.

For the outcome of spontaneous delivery prior to 32 weeks, we compared the Likelihood Ratios between the various models to determine the impact of adding the DOC and 16α-OHP to the model. The +LR for spontaneous preterm birth less than 32 weeks’ gestation based on the clinical covariates alone was 2.72 (95% CI 1.37, 5.39) for women with a positive test result. The addition of the DOC and 16α-OHP biomarkers to the model increased the +LR for spontaneous preterm birth less than 32 weeks’ gestation among women with a positive test result to 7.12 (95% CI 3.30, 15.39).

## Discussion and conclusions

Preterm delivery, particularly early in gestation, is strongly associated with neonatal morbidity and mortality. Our prior work demonstrated that maternal plasma levels of DOC and 16α-OHP, when analyzed during the late first trimester/early second trimester, were able to predict a woman’s risk for spontaneous preterm delivery prior to 32 weeks [5]. The current study demonstrates that DOC and 16α-OHP can also be utilized to predict the extent of preterm delivery-associated neonatal morbidity and length of neonatal hospitalization when measured early in pregnancy, prior to knowledge of any preterm labor symptoms or the gestational age at delivery. Knowledge of steroid administration and fetal sex allowed the neonatal model to improve its ability to predict neonatal morbidity, such that the baseline neonatal morbidity model could be updated later in pregnancy for women who develop symptoms that place them at risk for a preterm delivery. Further, regression modeling identified clinical and demographic factors known during the pregnancy that provided better predictive ability for spontaneous preterm delivery less than 32 weeks than the steroid biomarkers do alone [5]. This discovery work and findings need to be validated in an independent cohort before we can fully implicate the changes in these biomarkers with preterm birth and neonatal morbidity.

Very preterm delivery is a known risk factor for postnatal complications in the newborn, though not all very preterm babies experience postnatal morbidity. Aberrations in endogenous steroid concentrations have been independently identified as biomarkers detected from amniotic fluid for vePTD with and without neonatal bronchopulmonary dysplasia or death [8]. The current study demonstrates that maternal plasma biomarkers for vePTD and associated clinical variables measured in early pregnancy are predictive of the extent of subsequent neonatal morbidity and prolonged hospitalization. Our analysis suggests that there may be an inherently different endogenous steroid profile in pregnancies destined to end in preterm delivery with increased neonatal morbidity compared to those pregnancies with single or absent neonatal morbidity. This alternate explanation is supported by a study that used untargeted metabolomic analysis of amniotic fluid in the second trimester to identify pregnancies at risk of spontaneous preterm delivery and neonatal bronchopulmonary dysplasia [8]. Baraldi et al demonstrated that preterm deliveries complicated with bronchopulmonary dysplasia had a different amniotic fluid metabolic profile from those preterm deliveries without bronchopulmonary dysplasia. Leucinic acid, 4-hydroxy-3-methylbenzoic acid, 2-hydroxy caprylic acid, and 3-oxo-dodecanoic acid were differentially expressed in the amniotic fluid of babies delivering prematurely with bronchopulmonary dysplasia, while 3b,16a-dihydroxyandrostenone was differentially expressed in the amniotic fluid of babies born prematurely without bronchopulmonary dysplasia [8]. Their findings are very intriguing and are in line with our findings, suggesting that there are different metabolic or steroid profiles associated with preterm deliveries complicated by neonatal morbidity compared to those profiles associated with preterm deliveries and no neonatal morbidity. However, amniotic fluid is challenging to use for routine diagnostic or predictive testing, where our findings are based on maternal plasma testing obtained early in pregnancy. Similar to ours, their finding have also not been validated in another cohort.

Our study explored the potential of the maternal biomarkers DOC and 16α-OHP to predict pregnancies resulting in multiple major neonatal morbidities as measured with a composite neonatal morbidity scale (Hassan score ≥ 2). The study cohort had uniform maternal characteristics with the exception of BMI, while the neonatal characteristics were significantly different for many parameters (Table 1). While there was an expectation that some neonatal morbidity would be predicted due to the biomarkers’ association with occurrence of vePTD, the prediction of preterm delivery-associated neonatal morbidity was greater (AUROC 0.975) than that for vePTD alone (AUROC 0.94). Closer evaluation revealed that 4 of 9 subjects that had Hassan score ≥ 2 were from iatrogenic deliveries, with the remaining being spontaneous deliveries. Traditionally, spontaneous preterm deliveries and iatrogenic preterm deliveries were considered to have separate etiologies representing distinct molecular pathways. Iatrogenic preterm deliveries are largely attributed to the incidence of preeclampsia and/or poor fetal growth. In contrast, nine major etiologies have been proposed for spontaneous preterm delivery, with stress-related pathways as the most common etiology [4]. Though our initial findings demonstrate an ability to predict spontaneous vePTD [5], the findings of this study suggest that the maternal biomarkers DOC and 16α-OHP may be implicated in molecular pathways that lead to spontaneous and iatrogenic preterm deliveries early in gestation, with a common endpoint of increased neonatal morbidity.

A variety of “-omics” approaches (proteomics, genomics) have been used by other research groups to investigate similar endpoints [13–17]. Saade et al reported the ratio of two serum proteins, insulin-like growth factor-binding protein 4 (IBP4) and sex hormone-binding globulin (SHBG), measured between 19 0/7 and 20 6/7 weeks’ gestation, predicted spontaneous preterm birth less than 37 weeks’ gestation with an AUROC of 0.75 (75% sensitivity and 74% specificity) [13]. A follow-up study demonstrated that the serum ratio of IBP4/SHBG was able to predict preterm delivery less than 32 weeks’ gestation (AUROC 0.71, sensitivity and specificity not reported). Of the nine subjects in their cohort that delivered prior to 32 weeks’ gestation, only one delivered spontaneously while the other eight had medically-indicated preterm births [14]. The authors also demonstrated that the ratio of IBP4/SHBF could predict a composite neonatal morbidity score using the Hassan scale of 3 or greater (AUROC 0.67) [12, 14]. McElrath et al demonstrated that protein biomarkers from circulating microparticles could predict the risk for spontaneous preterm birth less than 35 weeks’ gestation (AUROC 0.74 with 70% sensitivity and 81% specificity) [15]. The current study, along with our group’s previous report, demonstrates that the maternal biomarkers DOC and 16α-OHP have the potential to advance the prediction of spontaneous preterm birth and neonatal morbidity to earlier in gestation [5].

Currently, the standard practice in clinical obstetrics is to determine a woman’s risk of preterm delivery based on a review of her obstetric history, though this approach misses over 90% of preterm deliveries [18]. A secondary approach of measuring the length of the cervix in the mid-trimester among low-risk women has become prevalent in the last decade, though it is not universally performed or predictive of preterm delivery [19]. The regression models developed in our study combine pathway-specific steroid biomarkers together with clinical variables known during the pregnancy that substantially improve the prediction of pregnancies at risk for early preterm birth and worsened neonatal outcomes. The development of improved screening tools that can be performed early in gestation is needed to identify the subpopulation of pregnancies that may benefit from increased prenatal surveillance or medical interventions. There is evidence that women with a history of preterm delivery managed in a prematurity prevention clinic have decreased rates of recurrent preterm delivery [20, 21]. In addition, recent data from Australia demonstrated that implementation of a comprehensive population-based, multifaceted program that was directed at reducing the rate of preterm birth within their government-funded universal health care system demonstrated decreased rates of preterm birth [22]. These prior studies suggest that execution of patient care models directed to women identified to be high risk for preterm delivery could result in improved outcomes by preventing preterm delivery.

Our study has several limitations. The Hassan score was utilized in our study as a simplified measure of composite neonatal morbidity. While there are alternate tools to assess neonatal morbidity with greater detail, the Hassan score allowed us to demonstrate the relationship between antenatal maternal biomarkers and neonatal outcomes using available clinical data. The relationship between the Hassan score as a measure of short-term neonatal morbidity and meaningful long-term outcomes has not been established and deserves further study. Another limitation is the small size of the cohort and the potential for overfitting. We attempted to address these limitations and mitigate this risk by optimizing the model’s AIC and R^2^ values with the least number of predictor variables to achieve model parsimony, but these approaches still may not account for all issues related to overfitting. In addition, as the initial sample size was limited, we were not able to conduct internal validation testing and generalizability of our findings to other populations should be approached with caution. As such, prospective evaluation of the regression models in a large pregnancy population is required to confirm our findings. Lastly, we chose to include only those variables known during the course of the pregnancy in the regression models to allow the tool to be a true predictive model of preterm delivery and adverse outcomes. The optimized neonatal morbidity model included whether women had received antenatal corticosteroids, allowing for adjustments to be made to the initial model that was developed early in pregnancy. Though antenatal corticosteroids clearly impact neonatal morbidity, at the time in the pregnancy that it is known that steroids are recommended, for some of these women little can be done to prevent delivery, limiting the clinical utility of the optimized model limited to predicting neonatal length of stay and morbidity events after the birth. We acknowledge that the neonatal morbidity findings associated with a positive screen may be largely driven by gestational age of delivery. Further, there may be additional clinical risk predictors not considered in our study that may influence the propensity towards very preterm delivery or neonatal morbidity.

In summary, our data demonstrate that the maternal steroid biomarkers DOC and 16α-OHP, when combined with patient demographic and obstetric data known during the course of the pregnancy, can accurately predict the risk of very preterm delivery and multiple major neonatal morbidity. The development of improved screening tools is a step towards early identification of pregnancies that may benefit from increased prenatal surveillance to prevent neonatal morbidity. The implementation of multimodal treatment pathways directed at women identified to be high risk may decrease the likelihood of preterm delivery. This is important as neonatal morbidities and prolonged neonatal length of stay are strongly associated with long-term handicaps and disabilities [23, 24]. As new screening approaches to estimate the risk of preterm birth are developed, it is important to focus on the associated neonatal outcomes as the true discriminator of clinical utility of such assessments.

## Supporting information

S1 Fig Initial neonatal morbidity model ROC curve, incorporating biomarkers and demographic and clinical characteristics. The initial neonatal morbidity model, which did not include whether antenatal corticosteroids had been given or fetal sex, demonstrated an area under the ROC curve of 0.927 (95% CI 0.824, 1.00) for discriminating subjects with a Hassan score of 2–4 (high neonatal morbidity) from those with a Hassan score of 0–1 (none or low neonatal morbidity). At this optimal cutoff value, the corresponding sensitivity was 90% and the specificity was 90%.
(TIF)
