# Supplementary figures and images for: Prediction of neonatal morbidity and very preterm delivery using maternal steroid biomarkers in early gestation
Source: PLoS One. 2021 Jan 6;16(1):e0243585. doi: 10.1371/journal.pone.0243585 (PMC7787372; doi:10.1371/journal.pone.0243585)

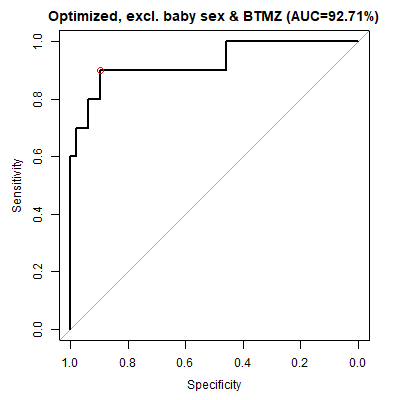

Supplement: S1 Fig — The initial neonatal morbidity model, which did not include whether antenatal corticosteroids had been given or fetal sex, demonstrated an area under the ROC curve of 0.927 (95% CI 0.824, 1.00) for discriminating subjects with a Hassan score of 2–4 (high neonatal morbidity) from those with a Hassan score of 0–1 (none or low neonatal morbidity). At this optimal cutoff value, the corresponding sensitivity was 90% and the specificity was 90%. (TIF) [file pone.0243585.s001.tif]
